# Estimating Crop Nutritional Status Using Smart Apps to Support Nitrogen Fertilization. A Case Study on Paddy Rice

**DOI:** 10.3390/s19040981

**Published:** 2019-02-25

**Authors:** Livia Paleari, Ermes Movedi, Fosco M. Vesely, William Thoelke, Sofia Tartarini, Marco Foi, Mirco Boschetti, Francesco Nutini, Roberto Confalonieri

**Affiliations:** 1 Department of Environmental Science and Policy, Università degli Studi di Milano, Cassandra lab, via Celoria 2, 20133 Milan, Italy; ermes.movedi@unimi.it (E.M.); fosco.vesely@unimi.it (F.M.V.); william.thoelke@unimi.it (W.T.); sofia.tartarini@unimi.it (S.T.); marco.foi@unimi.it (M.F.); 2 Italian National Research Council, Institute on Remote Sensing of Environment (CNR-IREA), via Bassini 15, 20133 Milan, Italy; boschetti.m@irea.cnr.it (M.B.); nutini.f@irea.cnr.it (F.N.)

**Keywords:** Critical nitrogen, NNI, PocketLAI, PocketN, sustainable N management

## Abstract

Accurate nitrogen (N) management is crucial for the economic and environmental sustainability of cropping systems. Different methods have been developed to increase the efficiency of N fertilizations. However, their costs and/or low usability have often prevented their adoption in operational contexts. We developed a diagnostic system to support topdressing N fertilization based on the use of smart apps to derive a N nutritional index (NNI; actual/critical plant N content). The system was tested on paddy rice via dedicated field experiments, where the smart apps PocketLAI and PocketN were used to estimate, respectively, critical (from leaf area index) and actual plant N content. Results highlighted the system’s capability to correctly detect the conditions of N stress (NNI < 1) and N surplus (NNI > 1), thereby effectively supporting topdressing fertilizations. A resource-efficient methodology to derive PocketN calibration curves for different varieties—needed to extend the system to new contexts—was also developed and successfully evaluated on 43 widely grown European varieties. The widespread availability of smartphones and the possibility to integrate NNI and remote sensing technologies to derive variable rate fertilization maps generate new opportunities for supporting N management under real farming conditions.

## 1. Introduction

Given the rising food demand and the need for reducing the environmental impact of cropping systems, increasing nitrogen (N) use efficiency is one of the greatest challenges the agricultural sector is facing [1]. After playing a key role in increasing yields during the Green Revolution, N fertilizers have progressively become a critical issue both for their frequent over-application and for the growing awareness of the negative impacts that agricultural activities can have on the health of agroecosystems [2]. Groundwater contamination, eutrophication, emission of greenhouse gases, and air pollution are negative externalities deriving from the improper use of N fertilizers, which led institutions to adopt specific measures to control N usage at the farm level (e.g., the EU Nitrate Directive 91/676/EEC). Moreover, the market is increasingly forcing farmers to minimize production costs, and fertilization—with N fertilizers playing the largest role—accounts for an important fraction of the total cost for input factors in both developed and developing countries (e.g., [3,4]). Another factor that strongly supports carefully optimizing N management is related to the negative effects of over-applications on many cereal species’ susceptibility to fungal pathogens [5] and lodging [6].

Long- and short-term strategies were proposed to increase nitrogen use efficiency. The former refers to the development of cultivars specifically improved for their effectiveness in uptaking and using N by exploiting traits related to root growth and activity, shoot N storage/remobilization, and leaf photosynthetic rates [7,8]. Among the short-term strategies, the adoption of variable rate (VR) fertilization appears to be a promising solution to reduce N losses and improve farmers’ income (e.g., [9,10,11,12,13,14]). However, estimation of the optimal N fertilization rate requires information on crop N nutritional status, in turn requiring the quantification of both N demand—via the concept of critical N concentration (Ncrit, % [15,16])—and actual N content in plant tissues [17,18].

In order to retrieve VR fertilization maps based on actual N nutritional status, spatially-distributed information on both plant N content (PNC, %) and the variables needed to estimate Ncrit (e.g., aboveground biomass or leaf area index) can be derived from satellite data, by means of empirical relationships between these variables and vegetation indices [19,20]. This process overcomes the limitations of cost and the low representativeness of direct field measurements. However, the estimation of biophysical variables from remote sensing data requires empirical relationships to be developed and validated using ground data [21,22], which can be effectively collected via smart scouting-driven field campaigns [23,24].

Different methods are available for indirect PNC estimates, ranging from complex instruments based on leaf spectral response, like Dualex (FORCE-A, Orsay, France) and SPAD (Konica Minolta, Tokyo, Japan), to simple approaches based on the visual evaluation of leaf colour (leaf colour charts [25]). All these methods indirectly estimate PNC under the assumption of a close relationship between leaf chlorophylls—and flavonoids in case of the Dualex—and N content in plant tissues. Commercial instruments are also available for non-destructive estimates of leaf area index (LAI, –), the driving variable of the non-destructive approach for determining Ncrit proposed by Confalonieri et al. [26]. Like the tools used to derive PNC, commercial instruments for indirect LAI estimates (e.g., LAI-2000, Li-Cor, Lincoln, NE, USA; AccuPAR, Decagon, Pullman, WA, USA) vary by complexity and cost. Although these instruments have demonstrated their reliability in a variety of studies (e.g., [27,28,29,30]), their effectiveness as operational tools in a farm context is limited by their low portability (especially those for LAI), their cost, and also by their expansive and time intensive maintenance in case of damages. Moreover, these instruments often lack internal systems for data processing and geo-referencing measurements, the latter being a crucial feature for precision farming applications. Another limit is that the information provided by instruments for different variables (e.g., PNC and LAI) cannot be integrated without exporting data to a personal computer, thus preventing their automatic integration for in vivo diagnosis of N nutritional status.

Besides technological aspects, an open issue for PNC estimates, and thus for N nutritional status determination, is that all indirect methods for PNC require dedicated calibration curves to convert readings (different types of indices according to the specific instruments) in PNC values, and these curves should account for genotypic variability, even within the same species [18]. Many authors, indeed, highlighted a cultivar-effect while using indirect methods to estimate PNC for different crops [31,32,33,34]. However, even though calibration curves for the main varieties grown in the area of interest are mandatory for the operational use of diagnostic methods for N nutritional status, their availability is strongly limited by the resources needed for their development and by the presence, in many production districts, of dozens of varieties differing in their colour response to N content in leaf tissues.

The objectives of this study were:

- Developing a smart app-based diagnostic system for supporting N fertilization via geo-referenced estimates of N nutritional status obtained by integrating apps for LAI (PocketLAI [35]) and PNC (PocketN [36]);
- Evaluating the system’s capability to estimate N nutritional status and to support N management through dedicated field experiments;
- Proposing an effective procedure to derive calibration curves for indirect methods for PNC estimates, and demonstrating its suitability in a case study with PocketN and 43 rice cultivars widely grown in Europe. This procedure would allow extending the diagnostic system to production districts where other varieties are grown.

## 2. Materials and Methods

Two field experiments were carried out in northern Italy in 2014 and 2015 with the aim of developing and testing the smart app-based diagnostic system to support rice topdressing N fertilization (experiments 1 and 2, hereafter). A third experiment was performed in 2017 in the same rice district to derive PocketN calibration curves for 43 widely grown European rice cultivars (experiment 3).

### 2.1. Experimental Design and Field Measurements to Develop and Test the Smart App-Based Diagnostic System for Supporting N Fertilization

Experiments 1 and 2 were carried out, respectively, in Rosasco (Pavia province; 45.27°N, 8.56°E, 114 m a.s.l.) in 2014 and in Gaggiano (Milan province; 45.24°N, 9.02°E, 117 m a.s.l.) in 2015.

In Rosasco (experiment 1), rice (cultivar Selenio) was row-seeded on 24 May 2014 (200 plants m^2^) and flooded at the 5th leaf stage, whereas in Gaggiano (experiment 2) cultivar Volano was scatter seeded (200 plants m^2^) on 6 May 2015 and grown under continuous flooding conditions. Rice was harvested on 16 September in Rosasco and on 11 September in Gaggiano. Soil was sandy-loam (USDA texture classification) in Rosasco, with low organic matter content (slightly above 1%) and low cation exchange capacity (CEC), whereas it was silt loam (USDA) in Gaggiano, with medium-high values for organic matter content (2.8%) and CEC. In both cases, available P and exchangeable K were not limiting, and pH was subacid. For both experiments, crop management allowed preventing water and nutrient (other than N) stresses and keeping the field pest-, disease-, and weed-free. Season 2014 was characterized by cold temperatures during summer and rainy conditions in late spring, which led to lower yields compared to the average [37], whereas during 2015, temperatures were lower than the average in the first part of the season and particularly favourable afterwards [38].

**Figure 1:**
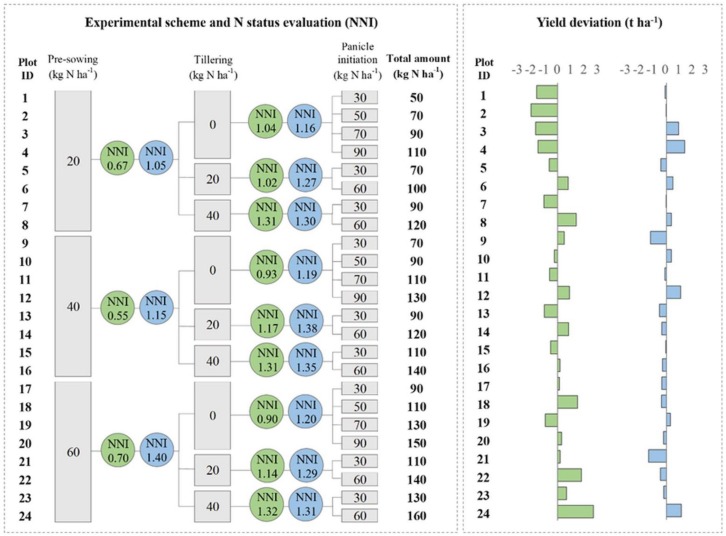
Experimental design (left panel) and yield deviations compared to the field mean (right panel) for the experiments performed in 2014 in Rosasco and in 2015 in Gaggiano (experiment 1 and 2 in the text, respectively). In circles: nitrogen nutritional index (NNI) values derived using the PocketN and PocketLAI smart apps just before the topdressing fertilizations at tillering and at panicle initiation.

The experimental scheme consisted of 24 fertilization storylines differing in terms of both timing (pre-sowing, beginning of tillering, and panicle initiation) and rating of N application (Figure 1), defined to reproduce the heterogeneity in N management options adopted in rice farms in the study area. Given the aim of evaluating N nutritional status under operational conditions, no “merely experimental” treatments—such as null or unlikely high N rates—were considered. Three N levels (20, 40, 60 kg·N·ha^−1^) were applied in pre-sowing to generate variability before topdressing fertilizations. At tillering (24 June in experiment 1 and 8 June in experiment 2), three N doses (0, 20, 40 kg·N·ha^−1^) were applied to each pre-sowing treatment to mimic farmers’ potential choices at this stage, including skipping this fertilization event to avoid excessive tillering and/or to reduce production costs. Fertilization at panicle initiation (15 July in experiment 1 and 29 June in experiment 2) is instead fundamental to guarantee high productivity and satisfying grain quality (e.g., [39]). Therefore, N was applied at this stage to each of the nine treatments (three N levels at pre-sowing × three N levels at tillering), with doses varying from 30 to 90 kg N ha^−1^. This led the overall seasonal N amount received by the 24 plots ranging from 50 to 160 kg·N·ha^−1^ (Figure 1). Nitrogen was always applied as urea, manually broadcasted on the plot surface. The size of each of the 24 plots was 30 m^2^ (3 m × 10 m).

Just before each topdressing fertilization event, PNC and LAI were estimated using the smartphone applications PocketN [36] and PocketLAI [35], respectively. Like for most of the instruments for indirect PNC estimates, PocketN derives PNC from leaf chlorophyll content, in turn estimated based on leaf greenness (*G*, –). According to Karcher and Richardson [40], the latter is calculated based on the hue, saturation, and brightness values (HSB colour space) of a 25-pixel leaf portion (Equation (1)):

(1) $$G=\frac{\frac{H-60}{60}+2-S-B}{3}$$

where *H*, *S*, and *B* are the values of hue, saturation and brightness.

In order to reduce the effect of lighting conditions on the smartphone camera settings, images were taken with leaves placed over a dedicated reference panel that flattened reflectance across the visible spectrum. The spectral behavior of the panel reproduced the one of the grey cards used in photography to get largely consistent exposure regardless of the light conditions. In particular, the PocketN panel reproduced the spectral behavior of the KODAK R-27 card (Eastman Kodak Company, Rochester, NY, USA). Contrary to the KODAK one, the PocketN panel was made of robust, washable plastic (expanded polyvinyl chloride) to avoid damages during field activities.

PocketLAI detects sky pixels (gap fraction) by automatically segmenting images taken at a 57.5° zenith angle [41], from below the canopy, while the user is rotating the smartphone along its main axis. The 57.5° angle is detected in real time by applying plain vector algebra to the components of the *g* vector as provided by the device’s 3-axis accelerometer. Gap fraction is converted into LAI values by inverting the Baret et al. [42] light transmittance model (Equation (2)):

(2) $$LAI=-\left[\frac{cos\left(57.5{}^{\circ}\right)}{0.5}\right]log\left[P_{0}\left(57.5{}^{\circ}\right)\right]$$

where *P_0_*(57.5°) is the gap fraction estimated at a 57.5° zenith angle. This model has been selected since it has been demonstrated that this particular view angle makes the information acquired independent from leaf angle distribution [42].

Further details on PocketLAI and PocketN usage are provided by Confalonieri et al. [35,36].

PocketN readings (unitless, ranging from 0 to 1) were taken at one third of the last fully unfolded leaf and converted into PNC values using the calibration curves for the cultivars Selenio and Volano, provided by Confalonieri et al. [36]. For both LAI and PNC, five readings were randomly performed for each plot, distributing the readings over the whole 30 m^2^ plot area. After PNC and LAI were estimated, 20 plants per plot [43,44] were also randomly sampled for determining aboveground biomass. At harvest, yield and aboveground biomass were determined from the dry weight of 20 randomly sampled plants and plant density.

In order to estimate N nutritional status, actual PNC was compared with Ncrit, which represents the minimum plant N content at which N does not limit crop growth. In particular, the nitrogen nutritional index (NNI [18]) was derived as the PNC to Ncrit ratio, with NNI values lower than 1 indicating N stress and values higher than 1 indicating luxury consumption [17]. Among the different approaches for estimating Ncrit, the MAZINGA model [26] was selected, given its higher feasibility for diagnostic purposes compared to approaches based on plant dry biomass determination [15] or numerical development stage codes (e.g., [45]). Being driven by LAI (estimated using indirect, non-destructive methods, like in this study), the MAZINGA model represents a real-time and cost-effective method for the quantification of Ncrit particularly suitable for operational contexts. According to the MAZINGA model, Ncrit is an inverse function of the fraction of radiation intercepted by the canopy (Equation (3)), indirectly representing the effect of leaf self-shading in remobilizing N from senescent tissues:

(3) $$N_{crit}=\frac{N_{mat}\;}{1-e^{-k\cdot LAI}}$$

where *Nmat* (%) is a parameter representing the value of Ncrit at maturity and *k* (–) is the extinction coefficient for solar radiation. *Nmat* and *k* were set here to 1% and 0.5, respectively [26].

The overall rationale of the experimental design was to generate plausible (i.e., coherent with management practices in the area) heterogeneity in N availability, in order to evaluate the suitability of smart app-derived NNI values as diagnostic information to support N fertilization. The evaluation was performed by analyzing the relationships between (i) the values assumed by NNI before each topdressing fertilization, (ii) the history of each plot (in terms of N fertilization), and (iii) the crop response, in terms of growth dynamics and final yield.

### 2.2. Definition of Calibration Curves for PocketN

A dedicated experiment was conducted in Gaggiano during 2017 (experiment 3) to derive PocketN calibration curves for 43 representative Italian rice cultivars (Table 1), selected because of their harvested area in the past three years (source: Italian National Rice Authority) and their potential relevance in the middle-term (information provided by seed companies). Some of these cultivars (or strictly derived ones) are also grown in other European countries and in the United States (e.g., Arborio). The 43 varieties were row seeded (row spacing: 18 cm; mean density: 200 plants m^−2^) on May 18, each in a 30 m^2^ (10 m × 3 m) plot and flooded at the 4th leaf stage. Field management allowed keeping the plots weed-, pest-, and disease-free, and without any nutritional or water stress. In particular, 135 kg N ha^−1^ were distributed as urea in three events: 25 kg·N·ha^−1^ at pre-sowing, 70 kg·N·ha^−1^ at tillering, and 40 kg·N·ha^−1^ at panicle initiation. In order to explore, for each cultivar, a wide range of PNC values, measurements were collected at five stages throughout the crop cycle (at the 3rd leaf, three tillers detectable, panicle initiation, flowering, and maturity) by exploiting the natural tendency of the plant to dilute N in aboveground tissues while growing [46]. After PocketN readings were taken (eight readings per plot), twenty plants per plot were randomly sampled for determining reference PNC values via elemental analyzer (model NA 1500, series 2, Carlo Erba, Italy). For each cultivar, reference PNC values were then related to the corresponding PocketN readings to derive cultivar-specific calibration curves via linear regression analysis. The analysis of the similarity among the parameters of the cultivar-specific curves was then used to cluster the cultivars. The assumption behind the clustering was that—in case of cultivars with the same colour response to N content—deriving curves specific for a group of cultivars would increase the robustness of the relationship between reference PNC and PocketN readings because of the higher number of data used. The k-means clustering method [47] was used, with the number of clusters defined iteratively targeting both the minimization of their number and the maximization of the R^2^ of the cluster-specific calibration curves.

## 3. Results

### 3.1. Evaluation of N Nutritional Status Via Smart Apps

Different pre-sowing N rates had a clear effect on crop growth in experiment 1, with aboveground biomass (AGB) at tillering in the amount of 0.40 t·ha^−1^, 0.48 t·ha^−1^ and 0.79 t·ha^−1^ for, respectively, plots that received 20, 40, and 60 kg N·ha^−1^ as basal N fertilization. Similar NNI values were achieved for all treatments at this stage (Figure 1), likely because larger N amounts turned into higher AGB accumulation rates and thus higher N demand. However, NNI highlighted a general situation of N stress (NNI < 1). In experiment 2, instead, similar growth rates were observed among treatments at tillering, with 20, 40, and 60 kg N ha^−1^ basal fertilizations leading to AGB values of 0.42 t ha^−1^, 0.43 t·ha^−1^, and 0.46 t·ha^−1^, respectively. Moreover, NNI values at the same stage (1.05, 1.15, and 1.40) increased with the amount of N distributed in pre-sowing (Figure 1), thus highlighting, in this case, unstressed conditions for N (NNI > 1).

**Figure 2:**
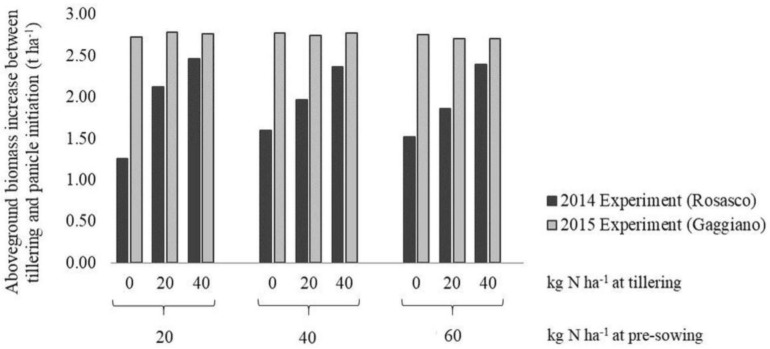
Response to different levels of topdressing fertilization at tillering in terms of aboveground biomass accumulation between tillering and panicle initiation.

The effectiveness of NNI in identifying unlimiting N conditions at tillering for experiment 2 was reflected in the limited crop response to different N levels distributed at the first topdressing fertilization (Figure 2). AGB accumulation between tillering and panicle initiation of plots that did not receive N was indeed basically the same as what was observed for plots fertilized with 20 kg N ha^−1^ and 40 kg N ha^−1^. In this case, the main effect of N applied at tillering was to level towards the top the NNI values at panicle initiation (Figure 1), as expected in the context of luxury consumption.

Rice, on the other hand, was highly responsive to different levels of N distributed at the first topdressing fertilization in experiment 1 (Figure 2), coherently with the overall conditions of N stress indicated by NNI (values lower than 1) (Figure 1). In this case, AGB accumulation after tillering in plots that received 20 kg N ha^−1^ or 40 kg N ha^−1^ was decidedly higher than for plots that received no N at this stage, especially for plots receiving 20 kg N ha^−1^ at pre-sowing (Figure 2).

In general, NNI values detected at panicle initiation remained lower in experiment 1 (Figure 1), although the plots that received the highest amount of N at tillering (40 kg·N·ha^−1^) showed similar NNI values in both experiments. This suggests for experiment 1 that only the highest N fertilization level at tillering allowed rice to recover from the N deficiency observed before the first topdressing fertilization.

The analysis of yield values for the plots with the same fertilization history until panicle initiation (e.g., plot from 1 to 4; Figure 1) allowed evaluating the relationships between NNI at that stage and the N amounts received at the second topdressing event. In experiment 2 (with NNI always higher than 1), a close relationship between the amount of N applied at panicle initiation and final yield was found: plots that received the highest N dose yielded more than the others (R^2^ = 0.40, *p*-value < 0.001). This relationship was even stronger for plots showing NNI values closer to one and fertilized with the lowest dose in the previous events ([plots 1-4], R^2^ = 0.92, *p*-value < 0.05), which largely relied on N applied at the second topdressing fertilization to satisfy their requirements. Plots showing the highest NNI values at panicle initiation (1.35 [plots 13 and 14] and 1.38 [plots 15 and 16]) instead revealed symptoms of luxury consumption (PNC was decidedly higher than Ncrit), with similar yields regardless of the amount of N received at the second topdressing event.

No relationship was found between yield and N applied at the second topdressing fertilization in experiment 1 (R^2^ = 0.01, *p*-value = 0.59), likely because the overall N stress occurred at the beginning of the season partly compromised the crop. This is clearly shown by plots 1–4, which presented NNI values around or lower than 1 before the two topdressing events. For these plots, crop potential was limited by insufficient N availability during the post-emergence and tillering phases, and—regardless of the N amount received at panicle initiation—final yields were decidedly lower than the field mean. This suggests that marked stresses during early stages can hardly be recovered with massive N fertilizations at panicle initiation, which in most cases would result in wasting N. In experiment 1, this is further demonstrated by the relationship between yields and total N received (R^2^ = 0.41, *p*-value < 0.001), with the highest productivity achieved by the plots that were less stressed during the first part of their cycle.

The variability in N treatments had a clear effect on rice productivity in experiment 1, with large differences in final yields among plots (the coefficient of variation was 26.8%; Figure 1). On the contrary, in experiment 2, the unlimiting conditions for N—highlighted by NNI values always above 1—resulted into a higher homogeneity in yields (the coefficient of variation was 9.9%, Figure 1) and in a lack of relationship between the yield and the total amount of N applied (R^2^ = 0.06, *p*-value = 0.23).

### 3.2. Calibration Curves for PocketN

**Figure 3:**
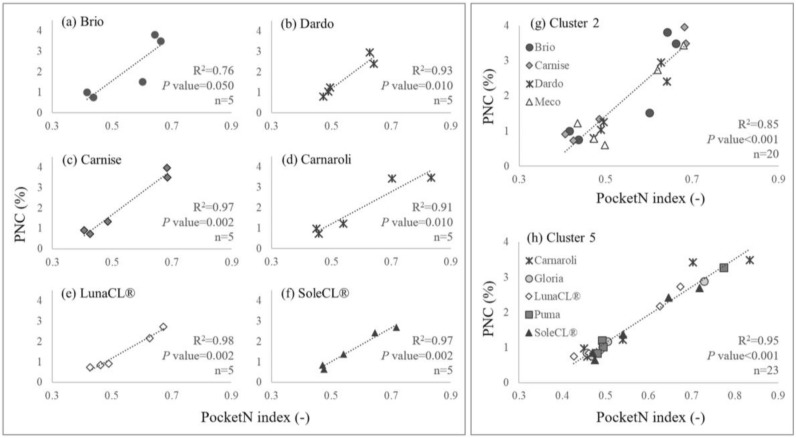
Examples (charts for all other calibration curves are available in Appendix A) of the relationships between the PocketN index (–) and reference plant nitrogen content (PNC) (%, from elemental analyzer) for six widely grown cultivars and for two groups of cultivars after clustering the calibration curves. See also Table 1 and Table 2.

The procedure used to derive PocketN calibration curves for the different rice cultivars allowed us to explore a wide range of PNC values, even without dedicated N treatments. Reference PNC varied from 0.60% to 4.22% (Appendix A), and presented the expected decreasing trend from post-emergence to maturity. Good agreement was found between PocketN readings and corresponding PNC values measured with the elemental analyzer (Table 1, Figure 3, Appendix A), with R^2^ ranging from 0.48 to 0.99 and mean R^2^ equal to 0.86 (R^2^ was higher than 0.70 in 35 out of 43 cases). The linear relationships used to convert the PocketN index into PNC were significant for most cultivars, with *p*-value <0.05 in 67% of the cases. For the cultivars for which the *p*-value was higher than 0.05, other regression models were tested, but no improvement in R^2^ and in significance level was obtained. Cultivar-specific calibration curves were largely heterogeneous (intercept varied from −33.8 to −0.1; slope ranged from 2.4 to 71.52; Table 1), highlighting, for rice, the importance of explicitly considering differences in the relationships between PNC and greenness among cultivars.

Eight groups of cultivars with similar color responses to N content in leaf tissues were obtained after clustering the cultivar-specific calibration curves according to the values of their parameters (Table 2, Figure 3, Appendix A). The R^2^ of the calibration curves derived for the clusters—ranging from 0.50 to 0.95—were fully comparable to those calculated for cultivar-specific relationships (Table 1). The clusters included 36 cultivars, whereas the remaining seven ones (i.e., Aiace, BaroneCL^®^, Cerere, Cleopatra, CRLB1, Keope, Selenio) could not be included in any of the clusters without a marked worsening of the relationship between PNC and PocketN readings. This suggested that we should adopt—for these cultivars—dedicated (cultivar-specific) calibration curves. As expected, the pronounced heterogeneity in cultivar-specific calibration curves turned into a large variability among cluster-specific ones (Table 2, Figure 3, Appendix A), with intercepts ranging from −7.03 to −1.24 and slopes between 5.4 and 17.2.

## 4. Discussion

The NNI derived from LAI and PNC measurements collected with smart apps proved to be effective in estimating N nutritional status. Conditions of N stress and luxury consumption were clearly detected, with NNI values lower than 1 in cases of N deficit and higher than 1 for well-fertilized plots. This was confirmed by the analysis of crop response to different N application rates, with direct relationships between AGB accumulation rates and N amounts in cases of N-stressed crops and, instead, similar growth rates for different N doses in cases of crops presenting N luxury consumption. This agrees with what reported by other authors that—although using other methods for estimating Ncrit and PNC—demonstrated the suitability of NNI for assessing N nutritional status [16,18] and, in turn, for supporting N fertilization (e.g., [48,49]) or for predicting yield variability [50]. The reliability of the NNI values derived in this study also proved the suitability of the approach used for non-destructive, LAI-based estimates of Ncrit. Moreover, this study confirmed the effectiveness of the PocketLAI and PocketN apps for quantifying LAI and PNC using widely spread and cost-effective tools like smartphones.

Both the experimental design and the choice of the two experimental sites (differing for soil texture and organic matter content) allowed testing the diagnostic system under a wide range of nutritional conditions, with rice experiencing different degrees of N stress in the first part of the season in experiment 1 and, instead, experiencing almost unlimited N availability in experiment 2.

In experiment 1, N stress was likely due to a combination of N leaching because of the sandy soil, low soil organic matter content, and the time lag between pre-sowing N application and the moment when the crop actually started uptaking N (the autotrophic phase). The retention capability of soils, indeed, can markedly vary according to physical and chemical soil properties [51]. For the same reason, the unstressed conditions for N (NN > 1) in experiment 2 can be explained by the silty-loam texture and the high organic matter content and CEC, which may have increased N availability [52]. Moreover, the low temperatures that occurred during early crop stages in experiment 2 could have lowered crop N demand because of reduced biomass accumulation rates. The overall unlimited N availability that characterised experiment 2 was clearly reflected in the closeness of yields resulting from different fertilization storylines compared to what achieved in experiment 1. This result agrees with what was reported by other authors, who observed lower heterogeneity at harvest in cases of unlimiting N conditions [53].

The peculiar design adopted for experiments 1 and 2 reflects the need to generate variability in N nutrition to test the diagnostic system in a range of conditions representative of operational contexts. For this reason, and because the aim was not to derive general conclusions on different products or fertilization strategies, we did not adopt a replicated standard design like those normally used to test the effect of factors like, e.g., alternative N fertilizers, doses, or timings. The aim was to test whether NNI values derived using smart apps were useful to make diagnoses of crop N status and to analyze them in light of the conditions affecting crop growth rates and productivity. Environmental (physical and chemical soil properties, as well as weather variables) and management (timings and amounts of fertilizers) conditions should be carefully considered, to properly interpret NNI values and effectively support in-season N management [54]. Our results showed, indeed, how the different conditions explored in the two experiments led NNI values assuming different meanings, thus limiting the possibility to directly use them to predict optimal N doses without interpreting the context NNI values refer to. Where environmental conditions favored low N use efficiencies (as in experiment 1), high N rates were required, even in the cases where NNI indicated a slight luxury consumption. On the other hand, where soil properties favored high N use efficiencies, high fertilization rates on plots where luxury consumption was detected (NNI > 1) led to waste fertilizer, since no relevant yield increase was obtained compared to lower N applications.

Calibration curves were derived in this study by exploiting the natural dilution of PNC along the crop cycle due to the relative increase of N-poor plant tissues (e.g., stems) and to the reallocation of N-rich compounds driven by leaf self-shading and senescence [46,55]. This strategy proved to be effective in exploring a wide range of PNC values, and allowed us to markedly reduce the effort needed to develop calibration curves compared to applying different N treatments. The explored range of PNC values was indeed comparable with those obtained in experimental designs with N doses as a treatment (e.g., [26]).

Given the role of the specific PocketN background panel in minimizing the impact of differences in light conditions, and the experimental design that allowed exploring a large variability in PNC and leaf greenness, the calibration curves obtained for the 43 varieties can be considered sufficiently robust for operational conditions. Although factors affecting leaf thickness may have an impact on the relationship between actual PNC and estimates from diagnostic tools based on leaf greenness [31], such an impact can be considered as negligible for supporting rice N fertilization compared to differences among cultivars. In this context, calibration curves derived from data collected in a single location can be considered robust enough (e.g., [56,57]).

Our results also underlined, once more, the within-species genotypic variability in terms of leaf properties (e.g., [34,58]), thus further highlighting the need to consider, explicitly, the heterogeneity among cultivars when using optical diagnostic tools for supporting N management.

**Figure 4:**
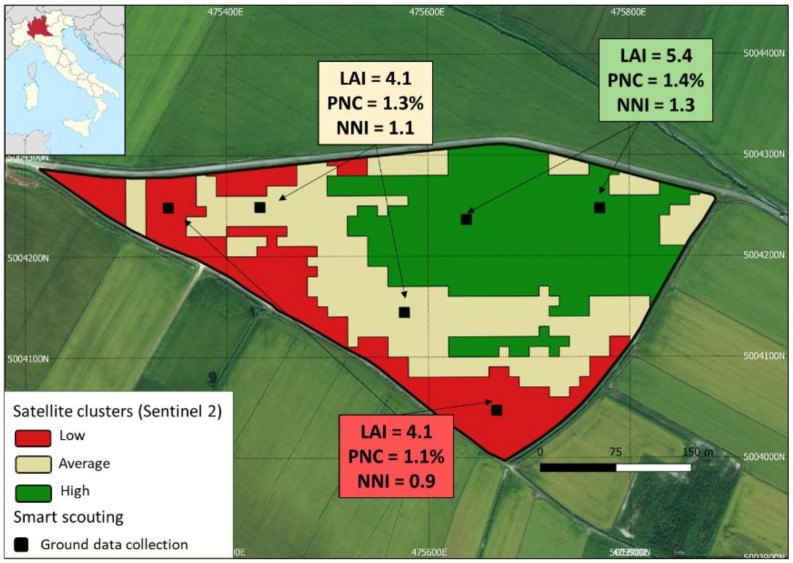
Example (for northern Italy) of the integration between satellite data and geo-located smart app-based N nutritional index (NNI) estimates. A cluster map from the 6 July 2018 NDRE (Normalized Difference Red-Edge) Sentinel 2 image was used to identify points where NNI was determined on 11 July (black boxes).

The possibility to estimate NNI using GPS-equipped devices that can be connected to back-end services allows the development of workflows that effectively exploit the integration of satellite data and the smart scouting-driven collection of agronomic information. Figure 4 shows an example from the SATURNO project (www.progettosaturno.it), aimed at providing farmers in Lomellina (northern Italy) N prescription maps. The within-field variability map was generated from the 6 July 2018 NDRE (Normalized Difference Red-Edge) Sentinel 2 image using k-means clustering according to Nutini et al. [24]. This clustering allowed the selection of locations in which to conduct six smart app measurements for analyzing the variability in N nutritional status. NNI was estimated using PocketLAI and PocketN, the latter using the specific calibration curve for cultivar Selenio, reported herein. These data—processed in a GIS environment—can support the definition of cluster-specific topdressing fertilisation. Moreover, empirical relationships between smart app data and satellite vegetation indices can be used to spatialize NNI over the whole field, thus supporting the development of variable rate N fertilization maps.

## 5. Conclusions

We developed a diagnostic system for supporting topdressing N fertilization and evaluated its reliability for paddy rice during two dedicated experiments. The system—estimating actual N requirements via NNI determination—targets operational farming contexts and is based on two smartphone applications for non-destructive estimates of Ncrit and actual PNC. The possibility of estimating NNI using inexpensive and highly portable tools that provide real-time information is expected to promote the adoption of the system within farming communities. According to the authors’ knowledge, this is the first time NNI has been derived and evaluated without using expensive and/or time-consuming methods, which could limit the potential for transferring NNI to operational farming contexts [18,50]. This system can be used as standalone tool or coupled to remote sensing technologies and smart-scouting techniques to derive NNI-driven variable rate fertilization maps [24].

PocketN confirmed its value as a diagnostic tool for non-destructive rice PNC estimates, regardless of the cultivar. Linear relationships between PocketN readings and PNC always explained a large part of the variance of reference values, with R^2^ very close to one, in most cases. Targeting the adoption of the system in operational contexts, calibration curves to convert PocketN readings in actual PNC values were derived for 43 popular European rice cultivars. According to the authors, this is the first time calibration curves were derived for a number of genotypes large enough to guarantee the applicability of non-destructive methods for PNC estimates in a production district. However, new tests could be needed for the varieties not included in any cluster, to provide further guarantees on the robustness of their calibration curves. The resource-efficient methodology used to derive the curves demonstrated the method’s effectiveness and could be exploited for other indirect methods (e.g., SPAD, Dualex) and cereal crops.

The obtained results and system applicability make this study an important step forward towards more mindful N management and, therefore, towards the improvement of the economic and environmental sustainability of farming activities.

## Figures and Tables

**Table 1:** Calibration curves, R^2^ and *p*-values derived from the PocketN index (–) and reference plant nitrogen content (PNC, %, from elemental analyzer) for the 43 rice varieties involved in the study.

ID	Cultivar	Calibration Curve Parameters ^a^		R^2^	*p*-Value	ID	Cultivar	Calibration Curve Parameters ^a^		R^2^	*p*-Value
		a	b					a	b		
1	Aiace	26.55	−11.53	0.87	0.060	23	Gladio	21.00	−8.47	0.98	0.010
2	Arborio	11.62	−4.23	0.70	0.160	24	Gloria	7.78	−2.78	0.99	0.003
3	Augusto	9.54	−2.95	0.81	0.040	25	Karnak	12.17	−4.21	0.66	0.190
4	Baldo	12.08	−4.44	0.97	0.002	26	Keope	23.70	−12.64	0.88	0.060
5	Balilla	7.45	−2.51	0.91	0.010	27	Leonardo	5.63	−1.04	0.78	0.050
6	BaroneCL^®^	24.67	−10.62	0.90	0.050	28	Loto	12.25	−4.98	0.94	0.010
7	Brio	10.70	−3.79	0.76	0.050	29	LunaCL^®^	8.32	−2.97	0.98	0.002
8	Cammeo	15.21	−6.81	0.95	0.020	30	MareCL^®^	9.63	−2.94	0.92	0.040
9	Caravaggio	8.75	−2.99	0.87	0.070	31	Meco	11.15	−4.28	0.85	0.030
10	Carnaroli	7.83	−2.71	0.91	0.010	32	Mirko	9.00	−2.69	0.48	0.200
11	Carnise	10.95	−3.80	0.97	0.002	33	Onice	6.61	−2.43	0.99	0.010
12	Carnise precoce	12.26	−4.92	0.96	0.004	34	Opale	5.61	−1.59	0.99	0.004
13	Centauro	5.50	−1.52	0.61	0.120	35	Puma	7.93	−2.86	0.99	0.010
14	Cerere	22.00	−10.23	0.60	0.230	36	Ronaldo	6.94	−2.15	0.97	0.020
15	Cleopatra	25.39	−13.64	0.63	0.200	37	Selenio	2.40	−0.09	0.63	0.210
16	CRLB1	71.52	−33.83	0.99	0.001	38	SirioCL^®^	11.71	−4.70	0.74	0.060
17	Crono	9.32	−3.23	0.99	<0.001	39	SoleCL^®^	8.34	−3.16	0.97	0.002
18	Dardo	10.94	−4.28	0.93	0.010	40	Thaibonnet	9.41	−2.69	0.83	0.030
19	Ellebi	6.29	−1.65	0.91	0.010	41	Ulisse	11.91	−4.69	0.67	0.090
20	Fedra	6.52	−2.39	0.99	0.005	42	Vasco	11.74	−4.28	0.79	0.110
21	Galileo	19.20	−8.46	0.99	<0.001	43	Volano	7.39	−2.41	0.96	0.020
22	Generale	15.36	−5.38	0.64	0.200						

^a^ Calibration curves defined as PNC = a × PocketN index + b.

**Table 2:** Rice varieties in each cluster and corresponding cluster-specific calibration curves to convert PocketN readings (–) into plant nitrogen content (PNC, %) values.

Cluster	Cultivars ^a^	Calibration Curve Parameters ^b^		R^2^	*p*-Value
		a	b		
1	Centauro, Ellebi, Leonardo, Opale	5.42	−1.24	0.76	<0.001
2	Brio, Carnise, Dardo, Meco	10.90	−4.02	0.85	<0.001
3	Galileo, Gladio	17.22	−7.03	0.83	0.002
4	Cammeo, Generale	9.97	−3.43	0.50	0.051
5	Carnaroli, Gloria, LunaCL^®^, Puma, SoleCL^®^	7.99	−2.87	0.95	<0.001
6	Augusto, Caravaggio, Crono, MareCL^®^, Mirko, Thaibonnet	9.04	−2.79	0.79	<0.001
7	Balilla, Fedra, Onice, Ronaldo, Volano	6.77	−2.25	0.91	<0.001
8	Arborio, Baldo, Carnise Precoce, Karnak, Loto, SirioCL^®^, Ulisse, Vasco	11.25	−4.19	0.79	<0.001

^a^ Aiace, BaroneCL^®^, Cerere, Cleopatra, CRLB1, Keope, and Selenio cultivars are not included in any cluster; ^b^ Calibration curves defined as PNC = a PocketN index + b.

**Figure A1:**
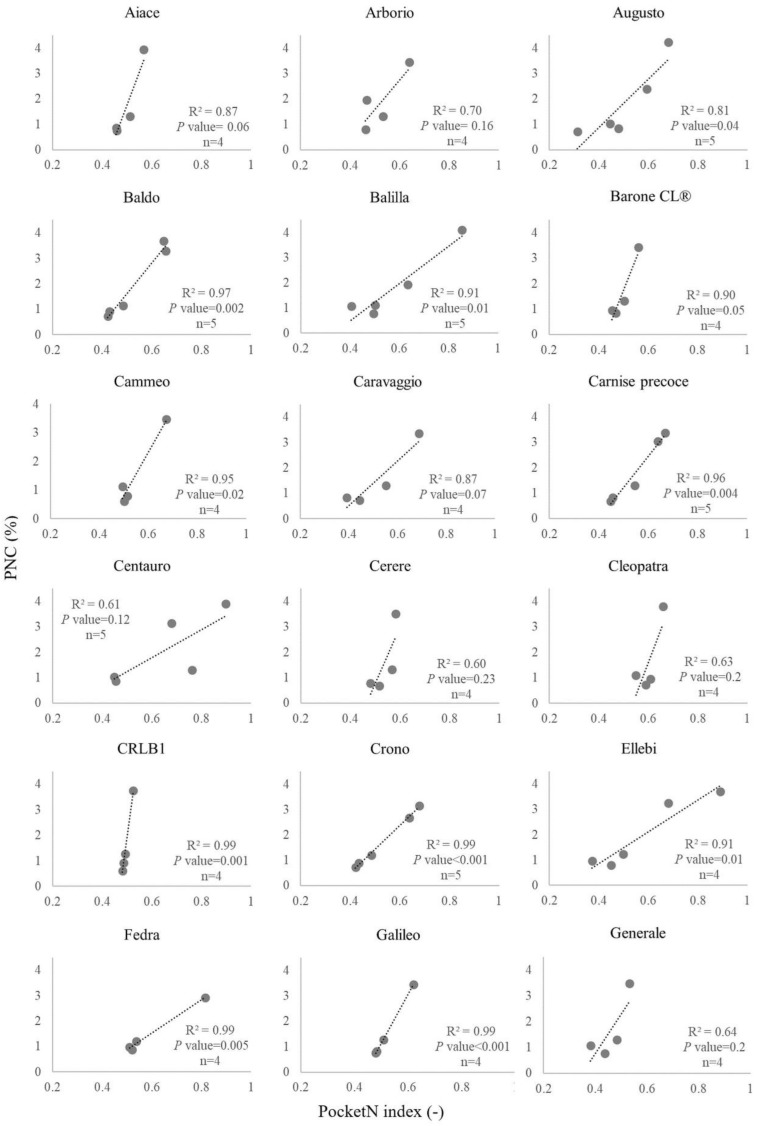
Relationships between the PocketN index (–) and reference plant nitrogen content (PNC) (%, from elemental analyzer) for eighteen rice cultivars included in the study. See also Table 1. These relationships were used to derive cultivar-specific calibration curves to estimate PNC using PocketN.

**Figure A2:**
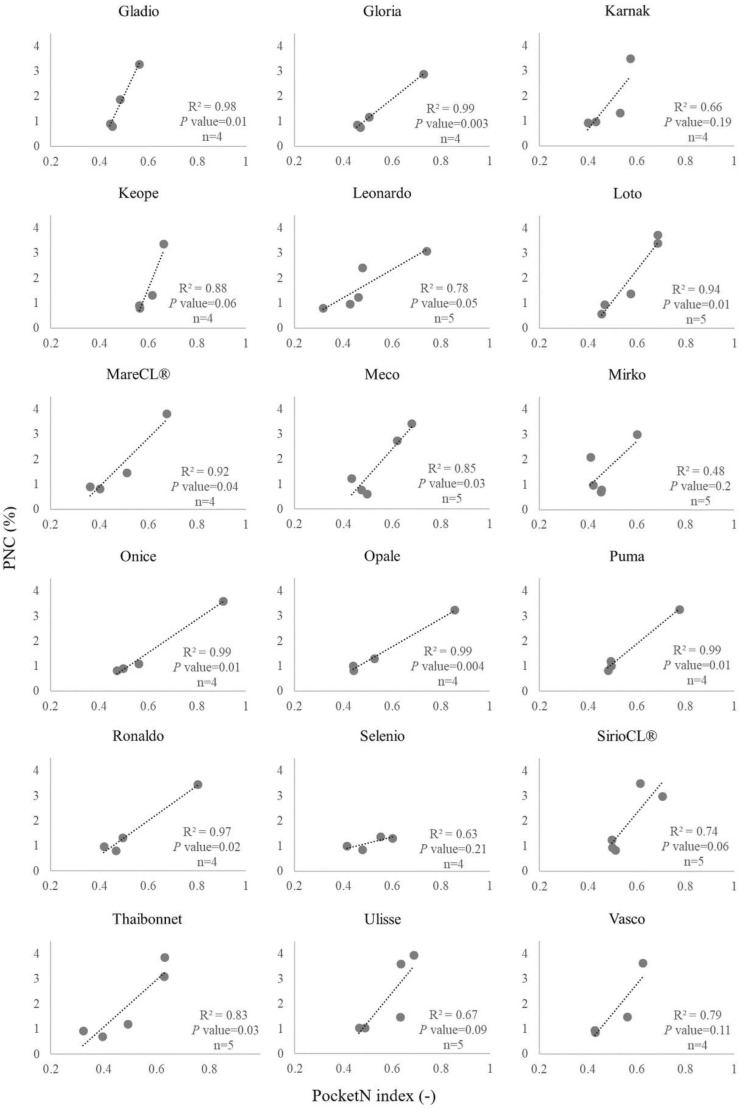
Relationships between the PocketN index (–) and reference plant nitrogen content (PNC) (%, from elemental analyzer) for eighteen rice cultivars included in the study. See also Table 1. These relationships were used to derive cultivar-specific calibration curves to estimate PNC using PocketN.

**Figure A3:**
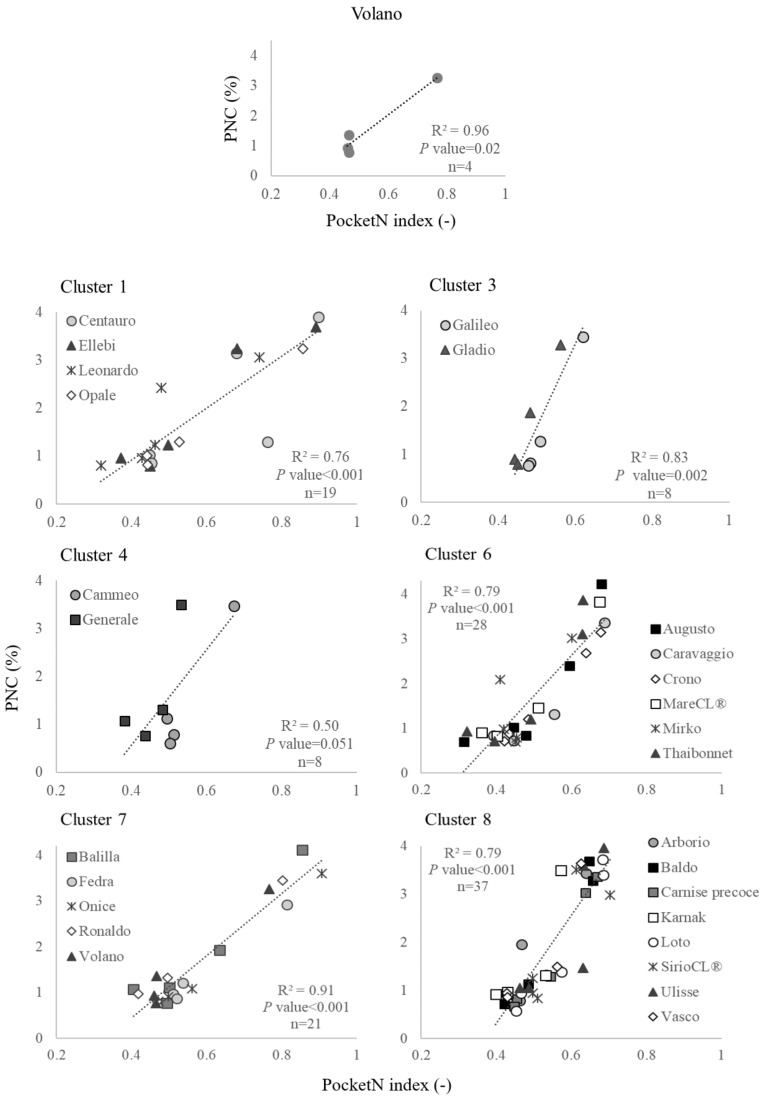
Relationships between the PocketN index (–) and reference plant nitrogen content (PNC) (%, from elemental analyzer) for the cultivar Volano and for cultivar clusters not included in Figure 3. See also Table 1 and Table 2. These relationships were used to derive cultivar-specific and cluster-specific calibration curves to estimate PNC using PocketN.

## Appendix A

Relationships between the PocketN index (–) and reference plant nitrogen content PNC (%, from elemental analyzer) for all the cultivars (Figure A1, Figure A2 and Figure A3) and cultivar clusters (Figure A3) except those shown in Figure 3. See also Table 1 and Table 2. These relationships were used to derive calibration curves to estimate PNC using PocketN.
